# Systematic Review on CyanoHABs in Central Asia and Post-Soviet Countries (2010–2024)

**DOI:** 10.3390/toxins17050255

**Published:** 2025-05-20

**Authors:** Kakima Kastuganova, Galina Nugumanova, Natasha S. Barteneva

**Affiliations:** Department of Biology, School of Sciences and Humanities, Nazarbayev University, Astana 010000, Kazakhstan; kakima.kastuganova@nu.edu.kz (K.K.); galina.nugumanova@nu.edu.kz (G.N.)

**Keywords:** cyanotoxins, Central Asia, CyanoHABs monitoring, water quality, Caspian seals, Pseudo-nitzschia, domoic acid

## Abstract

Cyanobacterial harmful blooms (CyanoHABs) in lakes, estuaries, and freshwater reser-voirs represent a significant risk to water authorities worldwide due to their cyanotoxins and economic impacts. The duration, spread, and severity of CyanoHABs have markedly increased over the past decades. The article addresses CyanoHABs, cyanotoxins, and monitoring methodologies in post-Soviet and Central Asian countries. This particular region was selected for the systematic review due to its relative lack of representation in global CyanoHABs reporting, particularly in Central Asia. The main aim of this systematic review was to analyze the primary literature available from 2010–2024 to examine the current situation of CyanoHAB detection, monitoring, and management in Central Asia and post-Soviet countries. Following a detailed database search in several selected data-bases (Google Scholar, Pubmed, Web of Science (WOS), Scopus, Elibrary, ENU, and KazNU) along with additional hand searching and citation searching, 121 primary articles reporting 214 local cyanobacterial bloom cases were selected for this review. Aquatic cyanotoxins were reported in water bodies of eight countries, including high concentrations of microcystins that often exceeded reference values established by the World Health Organization (WHO). Advancing monitoring efforts in Baltic countries, Belarus, and the Russian Federation differed from only a few Central Asian reports. However, Central Asian aquatic ecosystems are especially threatened by rising anthropogenic pressures (i.e., water use, intensive agriculture, and pollution), climate change, and the lack of adequate ecological surveillance. We hypothesize that recent Caspian seal mass mortality events have been caused by a combination of infection (viral or bacterial) and exposure to algal neurotoxins resulting from harmful algal blooms of Pseudo-nitzschia. We conclude that there is an urgent need to improve the assessment of cyanobacterial blooms in Central Asia and post-Soviet countries.

## 1. Introduction

Cyanobacterial harmful algal blooms (CyanoHABs) are a global concern, accounting for the majority of freshwater harmful algal blooms (HABs) worldwide [1]. These blooms pose serious threats to drinking and recreational water sources because many cyanobacterial taxa produce harmful hepatotoxins and neurotoxins [2,3,4,5]. HABs degrade water quality [6,7], lead to significant economic losses [8,9], and cause illness and mortality in wildlife, either through direct exposure to toxins or indirectly through the consumption of contaminated organisms, although these effects are often underreported [10,11]. Toxic CyanoHABs have been documented on every continent except Antarctica [12] and have been particularly recurrent in the Great Lakes region [3,13,14], as well as in various lake systems across the USA, Canada [15,16,17], China [18], and other countries. Over the past fifty years, the frequency and intensity of toxic algal blooms have significantly increased [19] across a range of trophic conditions [20]. A global analysis published in 2016 found that 108 countries reported cyanobacteria blooms associated with Microcystis [21].

On average, over 75% of CyanoHAB events are documented as toxic, indicating that most global water systems are likely to be contaminated with cyanotoxins [22]. Consequently, CyanoHABs greatly threaten water resources used for potable, recreational, and industrial purposes [23]. In recent years, there have been numerous instances of humans, domestic and wild animals becoming ill after drinking water contaminated with cyanotoxins [24,25]. Furthermore, when cyanotoxins accumulate in water sources, they are linked to incidents of liver, kidney, colon, and brain cancers [26,27]. Chronic exposure to these toxins is also associated with the development of neurodegenerative diseases [5,28,29,30,31,32]. On a global scale, the frequency of CyanoHABs is expected to rise due to climate change and the eutrophication of water resources [19]. As the occurrence of CyanoHABs rises worldwide, studying and constantly monitoring them with remote sensing can complement traditional CyanoHAB monitoring in making decisions in water quality management [33,34,35,36].

Extensive research has been conducted and published on the CyanoHABs, the distribution of cyanotoxins, and HAB-related diseases in South and North America, Asia, and Europe [37,38,39,40,41]. Systematic reviews have also examined trends in CyanoHABs within the Russian Federation and Baltic Sea countries [42,43,44,45,46,47]. However, reviews are rarely dedicated to coverage of CyanoHABs from Central Asia and post-Soviet countries. Central Asian countries frequently face significant challenges such as water scarcity, transboundary water issues [48,49,50], uneven usage of water resources [51,52], and increased deterioration of water quality due to industrial and agricultural waste [53,54,55,56]. Central Asia and the Middle East account for over 70% of the global net loss of permanent water bodies caused by climate change and anthropogenic impacts [57]. These water-related issues have intensified, leading to an uptick in local cyanobacterial blooms, resulting in massive fish kills and a rapid spread of toxic cyanobacteria in recreational waters [58,59,60,61,62,63].

This systematic review aimed to collect and analyze available research papers published between 2010 and 2024 on local cases of CyanoHABs and documented incidents of cyanotoxins, as well as the distribution of potentially toxic cyanobacteria in the fresh and brackish waters of Central Asia and post-Soviet countries.

## 2. Methods

This systematic review adapted the Preferred Reporting Items for Systematic Reviews and Meta-Analyses (PRISMA) guidelines. This review, however, did not include clinical data and, therefore, was not registered.

### 2.1. Search Strategy

A systematic review was conducted to explore underrepresented cases of cyanobacterial blooms and reported cyanotoxins in freshwater systems of Central Asia and post-Soviet countries. To perform this task, we narrowed down our keywords to the following ones: “country”, “freshwater”, “Microcystin”, “Nodularin”, “Anatoxin”, “CyanoHabs”, “cyanotoxins”, “cyanobacterial harmful algal blooms”, and “toxic cyanobacteria”. To refine the search results, we used Boolean operators where appropriate, following the next template, across all queries: “country” AND “freshwater” AND (“Microcystin” OR “Nodularin” OR “Anatoxin” OR “CyanoHabs” OR “cyanotoxins” OR “cyanobacterial harmful algal blooms”). The queries were performed using selected databases in English (Google Scholar, Web of Science, Pubmed, and Scopus), Russian (Google Scholar and Elibrary.ru), and Kazakh (Google Scholar, KazNU, and ENU repositories). The chosen period for publications was from 2010 to January 2024. In addition to the database searches, we identified 9 out of 121 studies through citation searching from the reference list of selected papers. The searches were carried out and assessed independently by two authors (K.K. and G.N.) based on the outlined eligibility criteria for inclusion.

### 2.2. Inclusion and Exclusion Criteria

**Figure 1:**
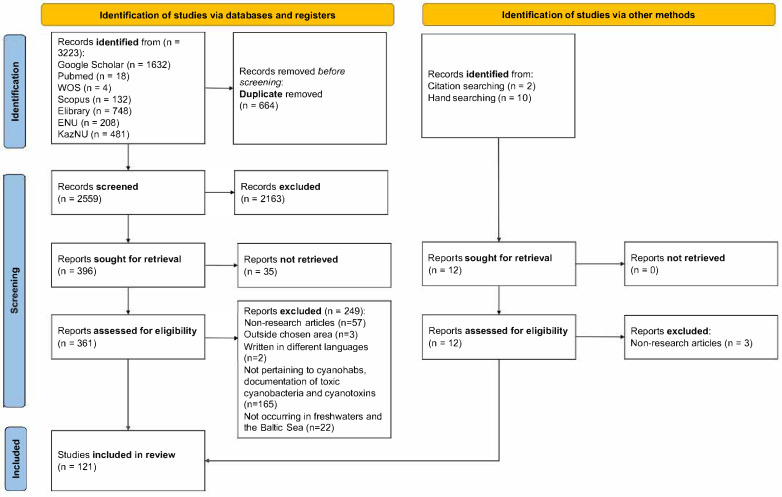
PRISMA flowchart for the systematic review summarizing the identification, screening, and inclusion stages.

Out of 3223 studies collected, only 121 studies were included in the systematic review (Figure 1). The studies were removed from the systematic review based on the following exclusion criteria: (1) the studies were not research articles (reviews, preprints, conference papers, book chapters, manuscripts, university materials, etc.); (2) the articles were devoted to CyanoHAB events occurring outside the chosen research area; (3) the articles were written in any language other than English, Russian, or Kazakh; (4) the articles were centered on any research topic other than CyanoHAB occurrence, cyanotoxins documentation, and toxic cyanobacteria; (5) the studies were focused on CyanoHABs in any water system except freshwater and the Baltic Sea; and (6) the articles were missing an available full text.

At each stage of screening, the articles proceeded to be included in the systematic review based on the following inclusion criteria: (1) articles documenting CyanoHABs, the presence of toxic cyanobacteria, and associated cyanotoxins in freshwater bodies; (2) articles focusing on the CyanoHAB situation in the Baltic Sea, included because of its closeness to freshwater systems (i.e., brackish water); (3) articles containing detection methods of cyanotoxins in freshwater systems of the chosen study area; and (4) articles including information on dominant and prevalent toxic cyanobacteria but with no prior detection of cyanotoxins. The process of selecting articles pertaining to the research question and the number of articles excluded at each step can be found in the flowchart (Figure 1).

### 2.3. Data Collection Process

The compiled articles were retrieved from the selected databases and later documented in Excel spreadsheets for duplicates and irrelevant studies. The stored articles were then used to address the following research questions: (1) What was the situation regarding CyanoHABs in Central Asia and post-Soviet countries? (2) What detection methods were employed in order to assess CyanoHAB events in freshwater bodies of Central Asia and post-Soviet countries? (3) Which toxic cyanobacteria species have been reported and implicated in the deterioration of water quality in these countries? (4) How can Central Asia and post-Soviet countries tackle the increasing threat of cyanobacterial blooming events in the future? The search across databases, including Google Scholar, PubMed, Web of Science (WOS), Scopus, Elibrary, ENU, and KazNU, yielded 3223 articles. After the removal of duplicates and screening of the title and abstract, 2559 articles were fully assessed for eligibility. Ultimately, 121 articles were included in the systematic review.

## 3. Results

### 3.1. Overview on Local CyanoHAB Events

**Figure 2:**
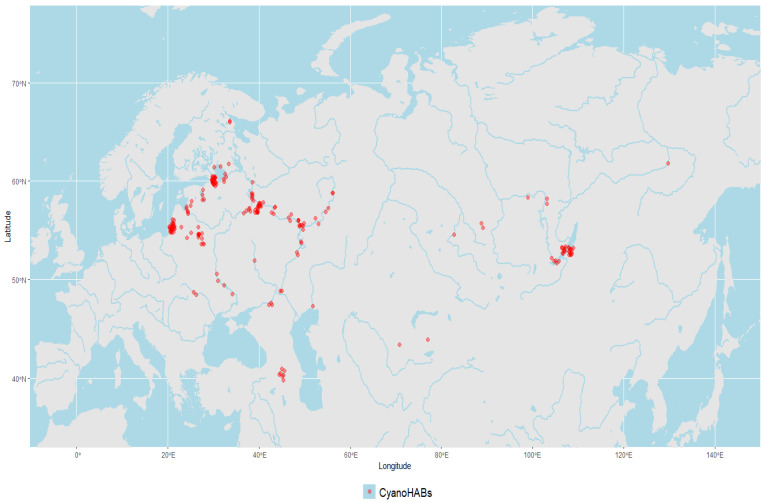
CyanoHAB events in Central Asia and post-Soviet countries.

The geographical locations of reported CyanoHAB events in Central Asia and former Soviet Union countries were used to build a map of local CyanoHAB events based on the information extracted from Table 1 (and Figure 2). Overall, 214 events of massive cyanobacterial blooms have happened in the fresh and brackish waters of Central Asia, former Soviet Union countries, and the brackish lagoon region of the Baltic Sea in recent years. Among these events are toxic algal bloom in the Kapchagay water body (Kazakhstan) (our unpublished data) and another in the Ural River [62].

### 3.2. Documented Presence of Cyanotoxins and Their Detection Methods

After reviewing the data, it is evident that a diverse range of methods has been used to analyze cyanotoxins, individually or in combination. Among these techniques, liquid chromatography–mass spectrometry (LC-MS) stands out, as it was utilized in 50% of the 68 analyses (Table 1). Additionally, molecular techniques, such as the polymerase-chain reaction (PCR), have been essential for detecting genes associated with toxin synthesis, along with antibody-based methods, like enzyme-linked immunosorbent assays (ELISA) [56]. In fact, more than 30% of the analyzed cases employed methods based on either the ELISA or PCR (Table 1).

The ELISA is an effective technique for routine water screening, capable of detecting total microcystin (MC) levels with high sensitivity and specificity. However, it cannot differentiate between different MC variants or assess their relative toxicity, and it may be significantly impacted by matrix effects [64].

High-performance liquid chromatography with UV detection (HPLC-UV) remains a classical method for cyanotoxin determination and is still used to detect MCs, nodularin (NOD), cylindrospermopsin (CYN), and anatoxin-a (ATX-a) [62,65], as shown in some articles from Table 1. Liquid chromatography coupled with tandem mass spectrometry (LC-MS/MS) provides higher selectivity, specificity, and sensitivity, which is critical for analyzing complex sample matrices that contain minute quantities of analytes. This elucidates its preference among some research groups for detecting cyanobacterial metabolites [66,67,68,69,70,71,72,73,74].

**Table 1:** Summary of local CyanoHABs, documented cyanotoxins, and their detection methods in water bodies of post-Soviet countries.

Location	Year/Period	Water Systems	Dominant Toxic Cyanobacterial Species	Cyanotoxin: Concentration (µg L^−1^, Unless Otherwise Specified)	Detection Method	References
Armenia	2012	Lake Yerevan	*Microcystis* spp., *Dolichospermum* spp., *Planktothrix* spp.	12 cyanotoxins including 10 MC congeners (max MC-RR)—0.1–34.8 µg g^−1^ dw); ATX-a—0.1–2.3 µg g^−1^ dw; CYN—0.1–0.3 ng mg^−1^ dw.	LC-MS/MS; ELISA (MCs)	[69]
	2018	Lake Sevan	*Dolichospermum* spp., *Aphanizomenon* spp., *Anabaena* spp., *Microcystis* spp.	40 cyanopeptide congeners (aeruginosins, microginins, ana-baenopeptines, cyanopeptolines, and 10 MC congeners)— Up to 2.5 μg L^−1^ (total).	LC-MS	[74]
	2020	Lake Sevan	*Microcystis* spp., *Aphanizomenon* spp., *Aphanothece* spp., *Dolichospermum* spp., *Anabaena* spp.		Biological testing	[75]
Belarus	2008–2010	Svisloch River	*Microcystis* spp., *Aphanizomenon flos-aquae*, *Anabaena* spp., *Planktothrix agardhii* *Aphanotece clathrata*	MCs (MC-LR, MC-VF)— 2.4 μg g^−1^ dw (total); Oscillamide Y—0.36 μg g^−1^ dw	LC/MS; ELISA, PCR (*mcyE*)	[76,77]
	2011–2012	River Viliya (Neris) and two tributaries: the Smerdiya and Usha Rivers	*Aphanizomenon flos-aquae*, *Dolichospermum* spp., *Microcystis* spp., *Planktothrix agardhii*	MCs (MC-LR; MC-WR; MC-RR, dmMC-LR; dmMC-RR)— peaks identified.	MALDI-TOF	[78]
	2012–2015	Lake Bol’shie Shvakshty	*Microcystis* spp., *Anabaena* spp., *Aphanizomenon flos-aquae*	MCs (MC-LR; MC-YR; MC-RR; dmMC-LR; dmMC-RR)—peaks identified.	MALDI-TOF; PCR	[79]
	2012–2016	35 fisheries	*Aphanizomenon* spp., *Anabaena* spp., *Microcystis* spp., *Oscillatoria* spp.		PCR (*mcyE*)	[80]
Estonia	2014–2015	Lake Peipsi	*Aphanizomenon* spp., *Dolichospermum* spp., *Microcystis* spp., *Planktothrix* spp.		Genus-specific qPCR (*mcyE*)	[81]
Kazakhstan	2016	Lake Bilikol	*Anabaena flos-aquae*, *Aphanizomenon flos-aquae*, *Microcystis aeruginosa*, *Oscillatoria* spp., *Phormidium tenue*, *Nostoc spp.*	MCs (MC-RR; 7-dmMC-RR; MC-LR)—peaks identified.	HPLC-MS; Daphnia test	[82]
	2019	Ural River	*Anabaena* spp., *Cuspidothrix issatschenkoi*, *Cylindrospermopsis ratiborskii*, *Dolichospermum* spp., *Pseudanabaena limnetica*, *Planktothrix* spp.	MCs, NOD—peaks identified; STX—ND.	UHPLC-DAD; PCR (*mcyE*, *sxtA*)	[62]
	2023	Kapchagai Reservoir		MCs—peaks identified.	UHPLC-DAD	Unpubl. data
Lithuania	2014–2015	Lithuanian Lakes: Gauštvinis, Jieznas, and Širvys	*Aphanizomenon* spp., *Cuspidothrix issatschenkoi*, *Sphaerospermopsis aphanizomenoides*, *Anabaenopsis cf.elenkinii*, *Cylindrospermopsis raciborskii*, *Dolichospermum* spp.	STX (Lake Jieznas)—up to 1.06 μg L^−1^); ATX-a (Lake Širvys)—up to 0.31 μg L^−1^; neoSTX, GTXs— peaks identified.	LC-MS/MS; PCR (*sxtA*)	[83]
Lithuania,	2014	Curonian Lagoon (southeastern Baltic Sea)	*Aphanizomenon flos-aquae*, *Microcystis* spp., *Planktothrix agardhii*	10 MC congeners— 0.52–153.60 μg L^−1^ (total); STX, ATX-a, and CYN—ND.	LC-MS/MS	[84]
	2013–2017	Curonian Lagoon (southeastern Baltic Sea)	*Aphanizomenon* spp., *Planktothrix* spp., *Microcystis* spp., *Dolichospermum* spp., *Woronichinia* spp.	27–34 cyano- metabolites at different sample stations; including 10 MC congeners, NOD, and ATX-a (detailed quantitative analysis).	LC-MS	[85]
	2018	Curonian Lagoon (southeastern Baltic Sea)	*Planktothrix agardhii*, *Microcystis* spp., *Aphanizomenon flos-aquae*	MCs confirmed.	Microcystin Strip test	[86]
	2018	Curonian Lagoon (southeastern Baltic Sea)	*Microcystis* spp., *Aphanizomenon* spp., *Dolichospermum* spp., *Planktothrix agardhii*, *Nodularia spumigena*, *Aphanocapsa* spp., *Limnococcus limneticus*	8 MC congeners—0.002–12.13 μg L^−1^; NOD—0.003–0.05 μg L^−1^; ATX-a—0.01–2.23 μg L^−1^.	LC-MS/MS	[87]
	2018–2020	Curonian Lagoon (southeastern Baltic Sea)	*Microcystis* spp., *Aphanizomenon flosaquae*, *Dolichospermum* spp., *Woronichinia compacta*	20 MC congeners—peaks identified.	LC-MS/MS; PCR (*mcyE*)	[72]
Russia	1999–2004 and 2005–2007	Lake Nero	*Planktothrix agardhii*, *Microcystis* spp.	MCs (MC-LR; MC-RR)— 0.55–12.91 μg L^−1^.	MALDI-TOF MS; HPLC-DAD	[88]
	2010–2011	Lake Nero	*Raphidiopsis raciborskii*, *Aphanizomenon gracile*	CYN—0.01–0.36 μg L^−1^.	LC-MS/MS; PCR (CYN biosynthesis genes)	[73]
	2000s	4 water reservoirs of Leningrad	*Planktothrix agardhii*, *Microcystis aeruginosa*	9 MC congener peaks identified.	HPLC	[89]
	2002–2008	Curonian Lagoon (southeastern Baltic Sea)	*Aphanizomenon flos-aquae*, *Anabaena* spp., *Microcystis* spp., *Planktothrix agardhii*		PCR (*mcyE*)	[90]
	2004–2005	Red Lake	*Anabaena* spp., *Aphanizomenon flos-aquae*, *Gloeotrichia echinulata*, *Microcystis* spp.	4 MC congener peaks identified.	HPLC-UV-MS; PCR (*mcyE*)	[91]
	2004–2005	Lake Ladoga	*Aphanizomenon* spp., *Anabaena* spp., *Anabaena affine*, *Microcystis* spp., *Woronichinia naegeliana*	5 MC congeners (MC-LR and others); 7 cytotoxins (anabaenopeptins and planktopeptin BL).	HPLC; biological tests (Daphnia)	[92]
	2004–2006	Beryozovskayartificial reservoir	*Aphanizomenon flos-aquae*, *Microcystis aeruginosa*		PCR (*mcyE*)	[93]
	2005–2012	Lake Baikal and water reservoirs of Angara water	*Anabaena* spp., *Aphanizomenon* spp., *Gloeotrichia echinulata*, *Microcystis* spp.	MCs; STX; neoSTX; GTX— 0.14–1.37 μg L^−1^ (total).	ELISA, LC-MS; PCR (*mcyE* and *sxtA*)	[94,95,96,97,98,99]
	2006	Curonian Lagoon (southeastern Baltic Sea)	*Microcystis* spp., *Aphanizomenon flos-aquae*, *Woronichinia compacta*	MCs—NA.	PCR (*mcyA*, *mcyE*, *mcyD*, *ana C*, *anaA*, *anaB*, *sxtA*, and *sxtI*)	[100,101]
	2006–2007	The Gulf of Finland (Baltic Sea)	*Microcystis* spp., *Anabaena* spp., *Woronichinia naegeliana*, *Gloeotrichia echinulata*	MC-LR; [DMAdda5]MC-LR; anabenopeptin F; micropeptin 88A; aerunogenosin 298A; anabaenopeptins; oscillapeptilid 97A; oscyllamid Y— peaks identified.	HPLC; biological tests	[102]
	2008–2010	Lakes Sestroretsky Razliv, Suzdal, Shchuchy, and the Gulf of Finland	*Planktothrix agardhii*	2010—MC-LR: 1.2–53.8 μg gr^−1^; MC-RR: 1.2–10.3 μg gr^−1^, ATX-a: <0.6 μg L^−1^.	LC-MS	[103]
	2008–2011	Sestroretsky Razliv	*Planktothrix agardhii*, *Aphanizomenon flos-aquae*, *Microcystis* spp.	MCs (MC-LR—0.02–0.2 μg L^−1^; dmMC-LR—0.02 μg L^−1^; MC-RR—0.01–0.09 μg L^−1^; dmMC-RR—0.01–0.04 μg L^−1^; MC-YR—0.01–0.02 μg L^−1^);—0.01–0.341 μg L^−1^ (total); ATX-a—0.8–5.0 μg L^−1^.	LC-MS	[104]
	2014–2018	Lakes Sestroretsky Razliv and Nizhny Suzdalskoye and the Gulf of Finland	*Aphanizomenon flos-aquae*, *Planktothrix agardhii*, *Microcystis* spp., *Planktolyngbya limnetica*, *Aphanocapsa* spp., *Woronichinia compacta*, *Dolichospermum* spp., *Aphanocapsa spp.*	20 MC congeners (detailed analysis by years/locations)— Up to 8.2 μg L^−1^ (Lakes); >40 μg L^−1^ (Gulf of Finland); ATX-a— 0.01–1.7 μg L^−1^.	HPLC-MS-HR	[105]
	2009–2011	Lake Nero and Upper Volga	*Planktothrix agardhii*, *Microcystis* spp., *Anabaena* spp.	MCs—NA.	ELISA; PCR (*mcyE*)	[106,107]
	2010	Lake Nero	*Cylindrospermopsis raciborskii*, *Planktothrix agardhii*, *Pseudoanabaena limnetica*, *Limnotrix redekei*	CYN—0.12–0.36 μg L^−1^.	LC-MS/MS	[60]
	2010	Rybinsk, Gorky, and Cheboksary reservoirs	*Aphanizomenon flos-aquae*, *Microcystis aeruginosa*, *Anabaena scheremetievi*, *Anabaena flos-aquae*, *Planktothrix agardhii*	9 MC congeners 0.079–8.375 μg L^−1^ (total).	LC-MS	[108]
	2010 and 2012	Kotokelskoe Lake	*Aphanocapsa* spp., *Anabaena* spp., *Microcystis* spp.	8 MC congeners— 13.8–76 μg L^−1^ (ELISA).	LC-MS; ELISA PCR (*mcyE*)	[109]
	2010–2012	Lakes of Saint Petersburg: Sestroretsky Razliv Lake (Razliv) and Lower Suzdal Lake (Suzdal)	*Aphanizomenon flos-aquae*, *Microcystis* spp., *Planktothrix agardhii*	14 MC congeners (Razliv)— 0.11–41.37 μg L^−1^; 9 MC congeners (Suzdal)— 0.01–2.89 μg L^−1^; ATX-a (Suzdal)—<0.54 μg L^−1^.	LC-MS	[110]
	2010–2012	Sestroretsky Razliv and Nizhny Suzdalskoye lakes	*Aphanizomenon flos-aquae*, *Anabaena* spp., *Microcystis* spp., *Limnothrix planctonica*, *Planktothrix agardhii*	MCs (MC-LR; MC-YR; MC-RR; D-Asp^3^-MC-RR; demethyl-MC-RR; MC-yR)—ext up to 0.211 μg L^−1^ ATX-a—ND.	LC-MS	[111]
	2011	Kuibyshev Reservoir and Rivers Kama and Mesha		MCs— 0.45–5.72 μg L^−1^.	ELISA	[112]
	2011	Kuibyshev reservoirs, River Mesha and Lake Nijnij Kaban	*Aphanizomenon flos-aquae*, *Microcystis aeruginosa*, *Anabaena* spp.	MCs (total)— 0.5–5.72 μg L^−1^.	ELISA	[113]
	2011–2013	Curonian Lagoon (southeastern Baltic Sea)	*Microcystis* spp., *Planktothrix agardhii*, *Aphanizomenon flos-aquae*, *Anabaena flos-aquae*	MCs—identified.	ELISA	[114]
	2012–2015	Sestroretsky Razliv	*Dolichospermum flos-aquae*, *Dolichospermum lemmermannii*, *Planktothrix agardhii*, *Aphanizomenon flos-aquae*	ATX-a—ND; STX, neoSTX, and GTXs—ND.	LC-MS; thiol-sensitive biosensors	[115]
	2012–2017	The Gulf of Finland (Baltic Sea)	*Aphanizomenon flosaquae*, *Planktothrix agardhii*, *Microcystis aeruginosa*, *Dolichospermum* spp.	9 MC congeners— Komarovo: ext up to 49 μg L^−1^; intra 466 μg g^−1^; ATX-a—ext 1.4 μg L^−1^.	HPLC-HRMS; genus-specific PCR (*mcyE* and *anaC*)	[116]
	2013	Rybinsk Reservoir	*Microcystis aeruginosa*, *Microcystis viridis*, *Planktothrix agardhii*, *Dolichospermum spp.*	MCs—1.7–5.8 μg L^−1^; STX—0.02–0.05 μg L^−1^; CYN, ATX-a—ND.	ELISA; PCR (*mcyE*, *anaA*, *anaC* and *sxtA*)	[117]
	2013	4 water reservoirs of Yaroslavl	*Microcystis* spp., *Aphanizomenon flos-aquae*	MCs—0.2–9.5 μg L^−1^.	ELISA; PCR (*mcyE*, mcyD, *anaA*, *anaC*, and sxtA)	[118]
	2013–2015	Sestroretsky Razliv Lake, Lower Suzdal Lake, Nero Lake, Rumnikovo Lake, Gorky Reservoir, Novosibirsk Reservoir	*Aphanizomenon* spp., *Microcystis aeruginosa, Limnothrix redekei*, *Dolichospermum* spp., *Planktothrix agardhii*	STX—intra 1.3–26.0 μg L^−1^, ext 174–1386 μg g^−1^ dw; ATX-a—3.0–35 μg g^−1^ dw.	LC-MS/MS; PCR (*sxtA, sxtI* and *anaC*)	[119]
	2013–2017	Volga River reservoirs, Curonian Lagoon (southeastern Baltic Sea), and lakes in the European part of the RF	*Microcystis* spp.	MCs—highly variable; 0.1–32.0 μg L^−1^ (total)	HPLC-HRMS; LC-MS/MS; ELISA; PCR (*mcyE* and *mcyD*)	[120]
	2017	Voronezhskoye Reservoir	*Microcystis* spp.	MCs (MC-LR; MC-RR; MC-YR)—19.73–88.68 µg L^−1^ (total).	HPLC-MS-MS	[121]
	2016	Mukhor Bay (Lake Baikal)	*Dolichospermum* spp., *Planktothrix* spp., *Aphanocapsa* spp.	MCs (MC-LA; MC-YR; MC-LF; MC-YM(O); dmMC-LR)— ext—1.2–3.39 μg L^−1^; intra 0.66–4 μg g^−1^ dw.	ELISA; LC-MS	[122]
	2016	Boguchansk water reservoir	*Aphanizomenon flos-aquae*, *Dolichospermum* spp.	MCs—0.3 μg L^−1^.	ELISA; PCR (*mcyE* and *sxtA*)	[123]
	2016	Lake Baikal	*Anabaena* spp., *Gloeotrichia echinulata*	MCs—0.11–6.2 μg g^−1^ dw.	ELISA	[124]
	2016 and 2018	Volga–Kama–Don water cascade	*Microcystis* spp., *Dolichospermum* spp., *Planktothrix agardhii*, *Aphanizomenon* spp., *Cuspidothrix issatschenkoi*, *Oscillatoria* spp.	14 MCs (MC-RR; MC-LR; MC-YR; dmMCs and others)— 0.1–16.4 μg L^−1^; ATX-a— 0.01 μg L^−1^.	LC-MS; HPLC-HRMS; PCR (*mcyE* and *anaC*)	[125,126]
	2017	Irkutsk Reservoir, 50 × 30 m water patch near hydroelectric dam	*Dolichospermum lemmermannii*	STX: HPLC-MS 600 ± 100 μg L^−1^; ELISA—2900 ± 900 μg L^−1^.	HPLC-MS; ELISA; PCR (*sxtA*)	[127]
	2017	Curonian Lagoon (southeastern Baltic Sea)		MCs—1–10 μg L^−1^.	Microcystin strip test	[128]
	2019	Lake Baikal	*Dolichospermum lemmermannii*	STX—ext 0.45 ± 0.05 μg L^−1^; Intra 7.900 ± 200 μg g^−1^ dw.	ELISA; PCR (*sxtA*)	[129]
	2018	Saint Petersburg water reservoirs		MC-RR and MC-LR peaks detected (NA).	HPLC-UV/MS	[130]
	2019	Svyatozero Lake	*Microcystis* spp., *Woronichinia naegeliana*	8 MC congeners— 6.22–6.34 μg L^−1^.	HPLC–HRMS	[131]
	2019–2020	Lakes Krivoe and Krugloe	*Dolichospermum lemmermannii*	4 MCs (MC-LR; MC-RR; 2 demethylated MC congeners) MC-LR—ext up to 78 ng L^−1^, intra—2 mg g^−1^ dw.	HPLC-HRMS	[132]
Ukraine	2017	Reservoir for Kasperivtsi Hydrothermal Power Plant, River Seret, and pond of Khmelnytsky Atomic Power Plant	*Cylindrospermopsis raciborskii*, *Aphanizomenon gracile*, *Dolichospermum flos-aquae*, *Planktothrix agardhii*, *Microcystis* *aeruginosa*, *Cuspidothrix issatschenkoi*	MC-LR; MC-YR; MC-RR; CYN; ATX-a—ND.	HPLC-DAD; Biological test	[65]

Abbreviations: ADDA—(all-S,all-E)-3-amino-9-methoxy-2,6,8-trimethyl-10-phenyldeca-4,6-dienoic acid; ATX-a—anatoxin-a; CYN—cylindrospermopsin; dmMC—desmethyl-microcystin; dmMC-LR—desmethyl-microcystin-LR; dmMC-RR—desmethyl-microcystin-RR; 7dmMC-RR—7-desmethyl-microcystin-RR; dw—dry weight; ELISA—enzyme-linked immunosorbent assay; ext—extracellular; GTXs—gonyautoxins; intra—intracellular; MCs—microcystins; MC-RR—microcystin-RR; MC-LR—microcystin-LR; MC-YR—microcystin-YR; MC-WR—microcystin-WR; MC-LA—microcystin-LA; MC-LF—microcystin-LF; MC-YM(O)—microcystin-tyrosine-methionine (oxidized); NA—not available (i.e., concentration was not reported, but qualitative analysis confirms the presence of a given cyanotoxin); ND—not detected; neoSTX—neosaxitoxin; NOD—nodularin; STXs—saxitoxins; UHPLC-DAD—ultra-high-performance liquid chromatography with diode array detector.

### 3.3. Dominant and Recurrent Potentially Toxic Cyanobacteria Genera

The identification of dominant cyanobacterial genera is important for monitoring bloom dynamics and managing CyanoHAB events. To identify the recurrent or dominant genera, information on potentially toxic cyanobacterial species was analyzed, counting the total number of mentions for each.

**Figure 3:**
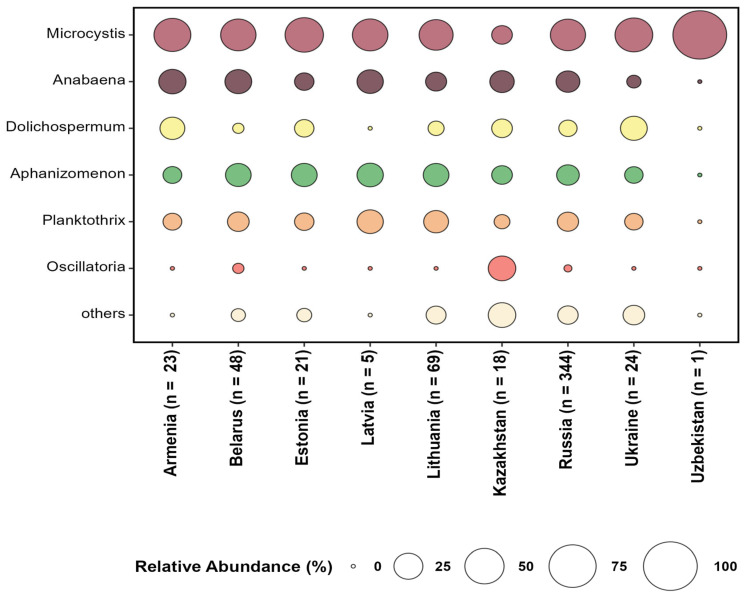
Dominant cyanobacterial genera detected in CyanoHAB events in water bodies of Central Asia and post-Soviet countries (n—total number of mentions of cyanobacterial genera for each country in the reviewed literature).

Notably, Microcystis, Anabaena, Aphanizomenon, Planktothrix, and Oscillatoria were frequently identified as the dominant genera causing cyanobacterial blooms in all countries. Information about CyanoHABs in Central Asian countries remains scarce, with only a few studies included in the systematic review (Figure 3). The cell counts of cyanobacteria were available only for some locations and time periods including Lake Bolshie Shvakshty [79], Lake Sevan [75], Lake Nero [88], Curonian Lagoon [90], Rybinsk Reservoir [117], Svyatoozero [131], and Sestroretsky Razliv [104,105]; in some cases, they significantly exceeded the threshold level (2 × 10^7^ cells L^−1^) for the safe recreational use of water bodies established by the World Health Organization (WHO).

## 4. Discussion

CyanoHABs are a worldwide problem, resulting in environmental adverse effects that have prompted the development of early detection and monitoring methods [133,134,135]. There are various in situ methods available to monitor and detect CyanoHABs [136,137], along with a range of advisory thresholds for managing harmful algal bloom impacts [138,139,140]. However, comprehensive field monitoring of CyanoHABs is challenging due to time, labor intensity, and costs. Evaluating CyanoHABs over extended periods and across large geographic areas is particularly difficult, compounded by issues related to sampling methodologies and the diversity of monitoring approaches [137]. Long-term studies on CyanoHabs have been conducted on multiple continents, providing valuable insights into their mechanisms and impact [141,142,143,144,145].

The guidelines regarding CyanoHABs primarily focus on cell concentrations rather than the levels of cyanobacterial toxins, since not all blooms generate toxins. In 1999, the WHO introduced a guidance level of 2 × 10^7^ cells L^−1^, which may warrant restrictions on the use of recreational waters [144]. Several countries have adopted this guidance as a basis for their action levels or guidelines, although there is considerable variation in cell count thresholds. According to the WHO classification, some water bodies described in the articles reviewed, such as Lake Bolshie Shvakshty (Belarus) [79], fall into the third hazard level and represent the highest risk to human health. This was also supported by data on cyanotoxin levels, which exceeded proposed WHO guidelines for both lifetime and short-term exposure to MC-LR through drinking water and exposure through recreational activities (1, 12, and 24 μg L^−1^, respectively) [139,143,144].

Significant progress has been made in developing satellite remote sensing methods to detect CyanoHABs in inland lakes [146,147,148,149]; however, these methods do not directly measure cyanotoxins [150]. Despite increasing awareness of CyanoHABs, there remains a notable underreporting of illnesses related to algae and cyanobacteria. This underreporting is mainly due to health facilities lacking the resources and expertise necessary to identify and monitor HAB events, as well as failure to collect and analyze environmental samples related to health accidents [35,151]. Human exposure to cyanotoxins occurs through different exposure routes, and there is a growing concern regarding the aerosolization and inhalation of cyanotoxins, though this area remains understudied [5,23,152,153,154,155,156].

The 121 articles included in the systematic review address the issue of CyanoHABs in Central Asia and former Soviet Union countries and document local cases of CyanoHABs occurring between 2010 and 2024. They also discuss the presence of potentially toxic cyanobacteria in their water systems and the methods used to detect cyanotoxins. According to the established inclusion and exclusion criteria, only articles dedicated to Armenia, Belarus, Estonia, Lithuania, Latvia, Kazakhstan, Russia, Ukraine, and Uzbekistan were analyzed in this review. However, Central Asian countries are often overlooked in the global coverage of CyanoHAB events [46,157,158,159,160]. The high number of publications on CyanoHABs describing the Baltic Sea region (specifically Estonia, Latvia, and Lithuania), as well as certain districts of the Russian Federation (including the Central, Northwestern, Volga, Siberian, and Far Eastern districts), can be attributed to established systems for the periodic monitoring of algal blooms. Additionally, a significant number of artificial reservoirs situated along major rivers contribute to CyanoHAB incidences [161,162,163]. The majority of articles originated from countries that are rapidly advancing monitoring efforts. The Baltic countries—Estonia, Latvia, and Lithuania—have adopted the European Union’s monitoring systems for HABs that include state-of-the-art technologies, such as the earth observation (EO) systems and periodic assessments using remote sensing, which help precisely locate the epicenters of blooming events [164,165]. Cyanobacterial blooms are reported and studied annually in many water bodies of Belarus [76,77,78,79,80] and the Russian Federation [88,89,90,91,92,93,94,95,96,97,98,99,100,101,102,103,104,105,106,107,108,109,110,111,112,113,114,115,116,117,118,119,120,121,122,123,124,125,126,127,128,129,130,131,132]. The minimum publications coming from Central Asian countries on cyanobacterial blooms may be explained not by the absence of CyanoHAB events but by the limitations of ecological monitoring.

While devising long-term strategies for managing CyanoHABs, two major challenges arise: changing climatic conditions and nutrient over-enrichment [134,166]. Climate change is a highly asymmetric but pressing issue for the world community [167]. The diversity of algal blooms and their impacts on water bodies make managing water resources particularly difficult. In Central Asian countries, including Kazakhstan, there has been a substantial increasing trend in the mean annual temperature over the past fifty years [168,169,170,171]. While nutrients, rather than temperature, are typically considered the main drivers of cyanobacterial biomass [159,160], water temperature may also play a crucial role in the proliferation of cyanobacteria, particularly in nutrient-rich eutrophic ecosystems [172,173,174,175,176].

The proliferation of CyanoHABs is driven by a complex interplay of abiotic and biotic factors. The cyanotoxins within biocrusts of drying lakes and deserts have started to be addressed recently [156,177]. Fluctuations in water levels, such as extreme rainfall and flooding events followed by dry and more physically stable conditions, promote the dominance of cyanobacteria [178]. Between 2002 and 2016, Kazakhstan experienced over 200 flooding events [179]; however, data scarcity hindered flooding risk assessment in Central Asian countries [180]. Moreover, CyanoHABs are stimulated by cultural eutrophication and excessive nutrient loadings in water reservoirs [40,167,181], mainly resulting from increasing inputs of nitrogen and phosphorus stemming from human sewage, livestock excrement, and synthetic fertilizers used in agriculture [182]. Irrigated agriculture is recognizable as a significant source of fertilizers and pesticides and significantly expanded in the 20th and early 21st centuries. In Central Asian countries, the use of mineral fertilizers significantly increased in the 2000s [183,184], and neighboring China ranks in the world’s highest position for fertilizer consumption. Considering the impact of HABs on freshwater bodies, it is essential to understand the distribution, biological effects, and occurrence of algal blooms and related toxins, particularly in regions where agriculture is projected to increase, such as the Ili-Balkhash Basin [185,186]. The Ili River region in Central Asia has not received the attention given to the Syr Darya and Amu Darya. However, the implications of recent dam and reservoir construction by China, as well as the long-term effects of Kapchagai dam and reservoir construction [187], and its possible links to fish kills in the region are of interest. The altitude (>3000 m) may limit the production of secondary metabolites and toxins by toxic algae [188,189]; however, among the included articles, no cases related to cyanobacteria blooms at high altitudes.

Though the Caspian Sea—the largest saline lake in the world—was outside the main topic of this review, it is another region of particular interest concerning CyanoHABs [190,191,192,193]. The ecosystem of this large, landlocked lake is undergoing dramatic changes, leading to a decline in all commercially important fish stocks, including all sturgeon species, Caspian herring (*Alosa capsica*), and anchovy kilka (*Clupeonella engrauloformis*), and a catastrophic decline in populations of Caspian seals (*Pusa capsica*) [194,195,196]. Since the 2000s, regular mass strandings of Caspian seals have been evaluated in the context of pollution, other anthropogenic factors, and infectious diseases, mostly viral [197,198,199,200]. However, HABs are among the leading causes of marine mammal mass mortality events (MMEs) [201,202], and exposure to low toxin doses over long periods may lead to a weakened immune system and increased susceptibility to viral and bacterial infections [203,204,205,206,207]. We hypothesize that Caspian Sea MMEs are caused by a combination of infection (viral or bacterial) and exposure to algal toxins resulting from HAB events.

Non-native *Pseudo-nitzschia serriata* from Bacillariophyta as well as another toxic species, *Nodularia spumigena,* from Cyanophyta present in high concentrations in the southwestern Caspian Sea [208], middle Caspian Sea [209], and the northern part of the Caspian Sea [210]. The Caspian seals migrate back to the north in late autumn for breeding [211]. Mass mortality events of Caspian seals periodically happened in late autumn (2022; 2024) and spring and were found to be related to viral infections [212,213]. However, there is currently no available data on deceased seals that have been examined for the algal toxins.

Pseudo-nitzschia blooms are prominent in northern Europe, and its toxigenicity is associated with domoic acid-producing strains [214]. However, the identification of toxigenic strains of Pseudo-nitzschia represents a chronic challenge for effective monitoring. Domoic acid exposure can be a plausible explanation for *Pusa capsica* MMEs because long-term toxin retention and higher trophic levels affect aquatic food-web dynamics [215]. Moreover, only Caspian seals and seabirds were affected in Caspian MME cases, and domoic acid-producing algal blooms do not cause fish kills [215,216]. Difficulties attributing causative agents to the Caspian Sea MMEs may be related to the to-tal overlooking of HABs and the absence of efficient ecological monitoring.

To develop reliable cyanobacteria and cyanotoxin monitoring system, several parameters have to be taken into account, including turnaround time, cost, and accuracy [138,217]. They may include microscopy [218], imaging and spectral systems, biosensors, NGS, ELISA, qPCR along with HPLC-UV, and LC-MS/MS systems data [62,219,220,221,222,223,224,225]. The limitation of UV-absorbance-based techniques is their susceptibility to background interferences, potentially leading to lower detection limits and false positive signals, depending on the specific matrices being analyzed [66]. In contrast, matrix-assisted laser desorption/ionization (MALDI), coupled with Time-of-Flight (TOF) mass spectrometry (MALDI-TOF), offers a soft ionization method for toxin analysis, even at the level of single colonies of toxic algae. Although TOF mass spectrometers are generally more sensitive than their counterparts, they are less commonly utilized for routine sample screening and quantitation compared to liquid chromatography–tandem mass spectrometry (LC-MS/MS) methods. The availability of high-resolution mass spectrometry (HRMS)-based instruments opened a possibility for the detection of low-molecular-weight compounds and their tentative identification in different degrees of confidence [67], which is of particular interest in the research of emerging toxins and those lacking analytical standards [68].

Some algal parameters can be quantified in real time and in situ, while algal toxins require laboratory access and a longer turnaround time. The microscopy method for cyanobacteria enumeration may require 2–5 days or more to obtain the results [218]. However, cyanobacteria’s doubling time can be significantly shorter, which makes risk assessment challenging. Monitoring activities may benefit from implementing multiple tools and providing com-plementary information. Using remote sensing and drones equipped with multispectral cameras can be important for developing early warning systems [34,226,227].

## 5. Limitations

The major limitation of this systematic review is selective reporting because of the outlined inclusion criteria and search strategy. Along with publication bias, some studies may not be included purely because of language differences (i.e., they were written in languages other than English, Russian, or Kazakh). Only selected databases were included for Kazakh (e.g., ENU and KazNU repositories), meaning that some research might have been overlooked by the reviewers. Frequently, the information regarding the presence of cyanotoxins in the waters of post-Soviet countries in the analyzed literature was limited, whereas in some articles, information about the cyanobacteria counts and taxonomic composition was absent. Given the data scarcity for the region, conclusions drawn from this review should be interpreted cautiously in light of the limitations.

## 6. Summary and Conclusions

A joint effort by transboundary state organizations and water monitoring agencies, combined with science-based legal frameworks, is required to reduce the current HAB-related threats in Central Asia. New policies based on an ecosystem-based approach have already been applied to CyanoHAB monitoring in different countries and continents, such as Australia, the United States, the European Union, and South America [228], and the WHO provisional guidelines provide threshold levels for some cyanotoxins (MCs).

Existing gaps in ecological monitoring can explain the limited available information about HABs in Central Asia and some post-Soviet countries. Thus, Caspian seals have been in rapid decline during the last decades, but the causes remain unknown. One factor potentially involved in the decline is the effect of algal neurotoxins on seals’ health and survival. *Pseudo-nitzschia* spp. are a part of the phytoplankton community in Caspian waters, and monitoring for domoic acid on a regular basis may be required.

To improve the assessment of toxic cyanobacteria blooms in Central Asia and post-Soviet countries, it is essential to adopt an intradisciplinary approach. This should involve early detection with remote sensing methods complemented by in situ evaluation of cyanobacteria and cyanotoxins using ELISA, PCR, and eDNA-based techniques. The analytical methods for cyanotoxin determination, such as HPLC-UV and high-precision LS-MS equipment, are sensitive and robust, albeit non-portable, and would require shipment of samples to centralized laboratories. Future long-term ecological monitoring, in parallel with fundamental research, requires the development of portable and affordable lab-on-chip kits and devices.

## Data Availability

No new data were created or analyzed in this study. Data sharing is not applicable to this article.
